# Population Effects of Influenza A(H1N1) Pandemic among Health Plan Members, San Diego, California, USA, October–December 2009

**DOI:** 10.3201/eid2202.150618

**Published:** 2016-02

**Authors:** Roger A. Bitar

**Affiliations:** Mission Infectious Disease and Infusion Consultants, Inc., at Palomar Medical Center, Escondido, California and Pomerado Medical Center, Poway, California, USA

**Keywords:** Influenza A virus, H1N1, 2009, pdm2009, influenza A(H1N1)pdm09, pandemic, viruses, population-based planning, healthcare delivery, intensive care units, telemetry, ventilation, oseltamivir, anti-infective agents, health planning, hospitalization, pandemics, pneumonia, inpatients, population dynamics, ambulatory care, antimicrobial, antiviral, vasoconstrictor, positive pressure respiration

## Abstract

Lacking population-specific data, activity of seasonal and pandemic influenza is usually tracked by counting the number of diagnoses and visits to medical facilities above a baseline. This type of data does not address the delivery of services in a specific population. To provide population-specific data, this retrospective study of patients with influenza-like illness, influenza, and pneumonia among members of a Kaiser Permanente health plan in San Diego, California, USA, during October–December 2009 was initiated. Population data included the number of outpatients accessing healthcare; the number of patients diagnosed with pneumonia; antimicrobial therapy administered; number of patients hospitalized with influenza, influenza-like illness, or pneumonia; level of care provided; and number of patients requiring specialized treatments (e.g., oxygen, ventilation, vasopressors). The rate of admissions specific to weeks and predictions of 2 epidemiologic models shows the strengths and weaknesses of those tools. Data collected in this study may improve planning for influenza pandemics.

## Abstract 2

Population-specific data were collected and analyzed to improve planning for influenza pandemics.

Planning for pandemic influenza would be enhanced by accurate prediction of the percent of the population that would be infected and those who would access healthcare; the level of outpatient and inpatient services, from primary to tertiary care; and the number of patients who had complications such as pneumonia and needed specialized care such as ventilation and observation in an intensive care unit. The pandemic of influenza A(H1N1) that occurred during 2009 (pH1N1) provided an opportunity to answer some of these questions and provide information that could assist in planning for future pandemics. Therefore, I conducted a retrospective study of members of the Kaiser Permanente (KP) health plan in San Diego, California, USA, who reported influenza-like illness (ILI) during the pH1N1 pandemic.

## Methods

### Patients and Study Design

This study does not identify the number of pH1N1 infections among the population but does identify the number of outpatients and inpatients in this population who accessed medical care. Data on outpatients, for whom influenza diagnostic studies were not done, includes the number who had ILI or influenza A and those who were treated with oseltamivir, received a diagnosis of pneumonia, were confirmed to have pneumonia based on chest radiograph, and were treated for pneumonia. Antimicrobial regimens administered are also documented. For inpatients, the data include the number admitted to a hospital with a diagnosis of ILI, influenza A, or pneumonia; those who were treated for ILI, pneumonia, or both; the antimicrobial regimens administered; the level of care received (regular medical or a higher level such as telemetry, assignment to an intensive care unit [ICU], bilevel positive airway pressure [BiPAP]/continuous positive airway pressure [CPAP], ventilation, vasopressors, and hemodialysis); the length of stay (LOS) in the hospital; and the results of testing for influenza A. These data are provided to assist medical and public health professionals in estimating the demand for outpatient and inpatient care and pharmaceutical supplies.

The members of the KP health plan are predominantly employed or formerly employed persons, which may mean that this population is not generally representative of the general population of the United States. However, it is similar to the general population in San Diego County (Technical Appendix Table 1).

Patient-specific data for KP members were extracted electronically from 2 sources: care provided to KP members by providers in the KP system and care provided by providers outside that system. For the months of October–December, 2009, the KP San Diego outpatient database was searched for all visits to a healthcare provider by persons with the diagnoses of ILI, influenza, or pneumonia; the inpatient database was searched for all discharges coded as ILI or influenza. Each of the electronic charts for outpatients that included diagnoses of ILI, influenza, or pneumonia was reviewed for documentation of a provider’s reading of chest radiograph, a radiologist’s report of chest radiograph, and treatment with antiviral or antibacterial therapy. Adhering to KP policy, nasopharyngeal swabs from outpatients were not sent for testing for influenza RNA by using PCR.

Electronic charts for inpatients that included ILI, influenza, or pneumonia were also reviewed for diagnosis of any of the 3 conditions and a reading of a chest radiograph by a provider and a radiologist. Also documented were treatment with antiviral or antibacterial therapy; level and length of care in medical, telemetry, or ICUs; receipt of respiratory therapy (oxygen, BiPAP/CPAP, ventilation); vasopressor therapy; hemodialysis’ LOS; and results of or lack of testing for influenza by culture, enzyme-linked immunosorbent assay (EIA), or PCR on secretions from a nasopharyngeal swab.

In addition, records of KP San Diego members who were seen by providers outside the KP system for whom influenza, ILI, and pneumonia were diagnosed were extracted electronically. For each of these patients, the LOS was available.

KP demographic data was electronically extracted from various databases. Annual median household income and education levels were determined on the basis of US Census Bureau–derived geocoding for the KP member’s ZIP code of residence (http://geocoding.geo.census.gov/geocoder). KP members were stratified into 3 groups (low, medium, and high) on the basis of the percentage of household members with a high school diploma or higher degree. 

Chronic conditions were extracted from a KP database that documents selected chronic conditions of particular interest to the health plan. Demographic data from San Diego County was supplied by an epidemiologist employed by the county (R.B.). Data on chronic kidney disease (CKD) was extracted from the United States Renal Data System based on data from the National Health and Nutrition Examination Survey (NHANES, http://www.usrds.org/atlas12.aspx).

### Case Definition

A case was designated as ILI or influenza on the basis of the provider’s diagnosis and the discharge diagnosis of the patient. Confirmation of the diagnosis of influenza was based on the results of a culture, an EIA, or PCR for influenza A RNA performed on nasopharyngeal swab samples. Diagnosis of pneumonia for all patients evaluated in a KP facility was based on a chest radiograph report by a radiologist, in contrast to diagnoses for patients evaluated in a non-KP facilities, which were based on the discharge diagnosis.

### Attack Rate

This study only provides data for the persons who accessed health care and does not include data for those who did not; thus, the attack rate in this population could not be calculated. Other studies have provided information on the attack rate. In 2010, Kelly et al. estimated the cumulative incidence of infection during the first wave of the 2009 pandemic as 16%–28% in preschool-age children, 34%–43% in school-age children, 12%–15% in young adults, and 2%–3% in older adults (*1*); the mean attack rate was 19.1%. Gilbert et al. estimated the attack rate of the 2009 influenza A(H1N1) pandemic to be ≈20.6% (*2*). The Centers for Disease Control and Prevention (CDC) published summary estimates of the morbidity and mortality of the 2009 pandemic during April 2009–April 2010 (http://www.cdc.gov/h1n1flu/estimates_2009_h1n1.htm); mid-level range estimates were ≈61 million cases for all ages. The population of the United States in 2009 can be estimated to be 306,013,175 based on the populations in the 2000 and 2010 census reports and the average incremental increase in the population, which was 2,732,363 per year. Using the estimate of ≈61 million cases for all ages and the population estimate of 306,013,175, the mean attack rate for all ages would be ≈19.9% (61 million divided by 306,013,175; http://www.census.gov/prod/cen2010/briefs/c2010br-01.pdf)

## Results

### Demographic Data

Complete demographic data consisting of age and sex distribution, race/ethnicity, language, estimated income, estimated education level, obesity, and smoking for San Diego KP members and for San Diego County residents are listed in Technical Appendix Table 1. Chronic conditions tracked among patients of KP and non-KP members in San Diego County are given in Technical Appendix Table 2, and the criteria that KP used to acquire the data are in Diagnostic Criteria in the online Technical Appendix. Combinations of selected chronic conditions in KP health plan members, as of December 2009, are provided in online Technical Appendix Table 3.

### Estimated Numbers of Infected Persons

On the basis of an attack rate of 20%, the estimated numbers of San Diego KP members with influenza A(H1N1) for October, November, and December 2009 were 99,144, 98,982, and 98,989, respectively (mean 99,038/3 months; Table 1). Of the estimated total San Diego KP health plan members infected, the numbers who accessed outpatient care during October, November, and December 2009 were 2,432, (≈0.49%), 3,202 (≈0.64%), and 1,038 (0.21%), respectively (Table 1). During those 3 months, pneumonia was diagnosed in 105 outpatients based on a radiologist’s report: 60 in the 0–18-year age group and 45 in the 19–>90-year age group. All were treated as outpatients: Online Technical Appendix Table 4 lists the number of persons with ILI, pneumonia by clinical diagnosis, and pneumonia by radiological diagnosis for age and gender. Table 1 lists by month the health plan population in San Diego, the estimated number of infections based on an attack rate of ≈20%, the number of members who accessed healthcare, the number diagnosed with pneumonia, and, for those evaluated in a KP facility, the number in whom pneumonia was diagnosed on the basis of a radiologist’s report.

**Table 1:** Estimated influenza-like illness among KP members treated as outpatients in KP and non-KP facilities, San Diego, California, USA, October–December 2009*

Characteristics	Total population					Rate per 100,000 members		
	October	November	December	Mean		October	November	December
KP health plan population, San Diego	495,718	494,911	494,947	495,192		100,000	100,000	100,000
Estimated infections (20% attack rate)	99,144	98,982	98,989	99,038		20,000	20,000	20,000
Number who accessed IPH	2,439	3,202	1,038	NA		492	647	209
Number of outpatients diagnosed with pneumonia	69	124	26	NA		13.9	25.1	5.3
Number of outpatients diagnosed with pneumonia by chest radiograph	31	57	17	NA		6.3	11.5	3.4
Number of outpatients diagnosed with pneumonia, OOHPC	23	17	18	NA		4.6	3.4	3.6
Number of outpatients diagnosed with pneumonia by chest radiograph, OOPHC	ND	ND	ND	NA		ND	ND	ND
Number of outpatients diagnosed with pneumonia	92	141	44	NA		18.5	28.5	8.9

*IPH, in-plan health care; KP, Kaiser Permanente; OOPHC, out-of-plan health care; NA, not applicable; ND, no data available.

The most frequently prescribed antimicrobial regimens for outpatients evaluated in a KP facility in whom pneumonia was diagnosed were azithromycin (n = 60) and amoxicillin (n = 47) for patients ages 0–18; for patients ages >18, moxifloxacin, doxycycline, and azithromycin were most frequently prescribed. Oseltamivir was administered to 104 (age 0–18) of ≈136 and 46 (age 19–>90) of ≈83 outpatients with pneumonia (values are estimates because age groups did not align exactly; Technical Appendix Table 5).

### Inpatients Infected

During October–December 2009, a total of 90 patients with ILI were admitted to a KP hospital: 34 in October, 52 in November, and 4 in December. Of these, 24 (26.7%) were 0–19 years of age and 66 (73.3%) were 20–­>90 years of age Technical Appendix Table 6). Nasopharyngeal swab samples for 55 of the 90 tested positive by PCR for influenza A; 2 tested positive by EIA and 1 by culture. No patient had a negative PCR and a positive EIA or culture (Technical Appendix Table 7). Seven patients were admitted to the ICU; 6 were placed on ventilators, and 5 were treated with vasopressors (Table 2). Inpatients with pneumonia indicated by chest radiograph had a longer LOS than did patients with ILI alone (Table 3).

**Table 2:** Number of inpatients with influenza-like illness who received specialized care at KP medical center, San Diego, California, USA, during October–December 2009*

Specialized care	No. patients	Total LOS, d	Mean LOS, d
Intensive care unit	7	126	18
Vasopressors	5	26	5.2
Ventilated	6	88	14.7
BiPAP/CPAP	12	26	2.2
Telemetry	17	161	9.5
Oxygen	51	ND	ND
Chronic hemodialysis	4	6	ND
Acute hemodialysis	5	51	10.2
Oseltamivir	87	ND	ND
Peramivir	1	ND	ND
Corticosteroids	12	ND	ND

*All 7 patients admitted to the ICU had positive PCR results for influenza A(H1N1)pdm09. Bipap, bilevel positive airway pressure; CPAP, continuous positive airway pressure; KP, Kaiser Permanente; LOS, length of stay; ND, no data available.

**Table 3:** Number of KP health plan members with ILI/influenza diagnosis only versus those with ILI and pneumonia, San Diego, California, USA, October–December 2009*

Characteristics	October	November	December
KP health plan population, San Diego*	495,718	494,911	494,947
Admitted to KP San Diego Medical Center, n = 90	34	52	4
Pneumonia diagnosis upon discharge	13	27	1
ILI/influenza	25	28	3
ILI/influenza, mean hospital LOS, d	4	3.5	2.7
Pneumonia based on chest radiograph	9	24	1
Pneumonia, mean hospital LOS, d	12.7	8.5	4
Admitted to non-KP hospital, n = 81	28	33	20
ILI/influenza	15	10	0
ILI/influenza, mean hospital LOS, d	2.5	3.2	2
Pneumonia	13	23	20
Pneumonia, mean hospital LOS, d	4.01	4.95	5.85
*Mean KP San Diego member population for October–December was 495,192. ILI, influenza-like illness; KP, Kaiser Permanente; LOS, length of stay.			

Of the 90 inpatients, 72 received antibacterial regimens (17 ceftriaxone/doxycycline and 13 ceftriaxone/azithromycin); 87 received oseltamivir (Technical Appendix Table 8). All 5 patients 0–18 years of age whose chest radiographs were read as pneumonia had positive PCR results for influenza A(H1N1)pdm09. Of the 16 whose chest radiographs were read as no pneumonia, 7 had positive PCR results and 7 were negative; PCR testing was not done for the other 2. Of those >19 years of age with pneumonia, 23 of 27 had positive PCR results. Of the 36 inpatients who did not have pneumonia, 18 were positive for influenza A by PCR and 16 negative; testing was not done for 2 (Technical Appendix Table 9).

## Discussion

Among the KP San Diego membership, a stable population, this study identified outpatients and inpatients during October–December 2009 who were diagnosed with influenza or ILI. All outpatients with a clinical influenza/ILI diagnosis, and those with that diagnosis and pneumonia, and the antimicrobial regimens prescribed, were recorded. Inpatients with clinical influenza/ILI diagnosis, with that diagnosis and pneumonia and the level/intensity of care rendered were recorded. In addition, tests for influenza A and the antimicrobial regimens were logged. This combination of data provided a comprehensive profile of these patients with influenza/ILI. However, neither the number of patients in this population with influenza A(H1N1)pdm09 nor the attack rate could be determined.

This study accepts an estimated attack rate of ≈20%. Estimates such as these are useful when planning for pandemic influenza; however, I found no studies that logged the number of visits to outpatient healthcare in a specific population, as this study does. For a monthly health plan population of ≈495,000, an attack rate of 20% would have resulted in ≈99,000 cases of influenza A(H1N1)pdm09 per month, but only a small percentage of the estimated number of infected persons accessed medical care. During October, November, and December, 2,432 (2.5% of estimated infected), 3,202 (3.2% of estimated infected), and 1,038 (1.0% of estimated infected) outpatient visits were recorded, respectively (Table 1). 

As part of this study, I reviewed the demand versus supply of antimicrobial agents prescribed to outpatients. During October–December 2009, a total of 6,672 KP patients with ILI accessed outpatient care; 219 had diagnoses of and were treated for pneumonia. Of those 0–18 years of age, 64 received azithromycin. The hospital’s 1-day par level (minimum in-stock quantity) was adequate for 117 patient-courses of 100 mg/5 mL, 361 patient-courses of 200 mg/5 mL, and 2,990 of 250 mg. Of those age >19 years of age, 16 received azithromycin 500 mg, requiring 96 tablets, versus a 1-day par level adequate for 1,309 patient-courses. Of those >19 years of age, 10 received amoxicillin, requiring 10 days or 100 tablets; the 1-day par level as adequate for 3,117 patient-courses of 250 mg and 17,503 of 500 mg. Par levels given are from 2014, when monthly population was approximately the same as in 2009.

The rates of ILI admissions per 100,000 KP members during October, November, and December 2009 were 12.5, 17.2, and 4.8, respectively. These rates represent 2.5%, 2.6%, and 2.3% of outpatient visits, respectively (Table 1). 

In 2005, the CDC published the FluSurge 2.0 software program, which is a tool for projecting the number of hospitalizations, ICU admissions, patients requiring ventilation, and an estimated mortality rate that might be anticipated in medical facilities during a pandemic (http://www.cdc.gov/flu/pandemic-resources/tools/flusurge.htm [*3*]). In November 2009, the CDC published the FluSurge Special Edition, specifically tailored to the 2009 influenza A(H1N1) pandemic (http://www.cdc.gov/h1n1flu/tools/flusurge/). These programs project admissions for 3 scenarios during an influenza A pandemic: minimum, likely, and maximum.

For the San Diego KP membership, rounded to 500,000, Technical Appendix Table 10 contains the data for the most likely scenarios projected by FluSurge2 (designated FluSurge05) for an attack rate of 15% and the FluSurge Special Edition (designated FluSurge09). The data include the distribution of patients admitted to a hospital, treated in ICU, and ventilated and those who died per week predicted by these programs. A comparison of the FluSurge predictions by these programs for the number of admissions to the hospital for the KP population versus the actual number of members admitted to the hospital shows that the minimum estimated number of admissions/week by the FluSurge05 program (attack rate 15%) and the most likely estimated number of admissions by the FluSurge09 program was approximately the same as the actual number of admissions/week in this study at an attack rate of 20% (Figure). If one accepts the San Diego KP population as a fair approximation of the general population in San Diego County (Technical Appendix Table 1), the FluSurge09 program demonstrates substantial improvement in the ability to predict the number of admissions to the hospital over FluSurge05. However, for the data in this study, the most likely scenario projected by the FluSurge09 program overestimates the number of patients projected to require ICU care by 1.6 times and ventilation by 1.3 times, although these estimates are still an improvement on those from FluSurge2 (attack rate 15%), which overestimates the number requiring ICU care by 3.5–7-fold and the number projected to need ventilation and admission by ≈3-fold (data not shown). Baker et al. also found that the FluSurge2 most likely scenario overestimated the number of persons projected to require admission, ICU care, and ventilation (*4*). The FluSurge2 projection of the number of patients on ventilators was a factor that influenced KP California to buy additional ventilators for stockpiling (Kaiser Permanente, unpub. data). The number stockpiled may have been fewer if it were not for the FluSurge2 projection. However, so that adequate surge policy is adopted and adequate supplies stockpiled, it may be beneficial for the estimates to be higher than that required, although over-stockpiling may not be cost-effective.

**Figure:**
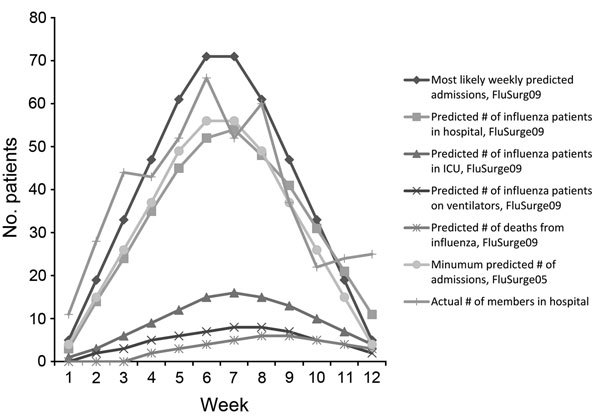
Hospital admissions per week for a predicted Kaiser Permanente health plan population of ≈500,000 members versus actual numbers of inpatients admitted to Kaiser Permanente San Diego Medical Center during the influenza A(H1N1) pandemic, San Diego, California, USA, October–December 2009. Predictions were compiled by using FluSurge2 (FluSurge05) and FluSurge Special Edition (FluSurge09) (http://www.cdc.gov/flu/pandemic-resources/tools/flusurge.htm) software Assumptions for FluSurge2: average length of non-ICU hospital stay for influenza-related illness, 5 d; average length of ICU stay for influenza-related illness, 10 d; average length of ventilator usage for influenza-related illness, 10 d; average proportion of admitted influenza patients who will need ICU care, 15%; average proportion of admitted influenza patients who will need ventilators, 7.5%; average proportion of influenza deaths assumed to be hospitalized patients, 70%; daily percentage increase in cases arriving compared to preceding day, 3%; attack rate, 15%; total no. hospital beds 392, ICU beds 34, ventilators 40. Unable to find assumptions for FluSurge Special Edition. ICU, intensive care unit.

One death occurred among the 90 inpatients during the 3-month study period. A previous study of a selected population of 108 patients categorized as having moderate to intermediate illness related to diagnoses of influenza A(H1N1)pdm09 found no deaths, although that study excluded patients requiring ventilation (*5*). In a United States study, Skarbinski et al. reported a death rate of 8% among 255 inpatients in 45 states (*6*). For a 15% attack rate, the FluSurge2 program predicts 8 and 31 deaths, respectively, in weeks 4 and 8 during an influenza pandemic, and the FluSurge Special Edition predicts 2 deaths at week 4 and 6 at week 8.

In this study, 47 inpatients received ceftriaxone, the most frequent antibacterial agent ordered. The daily par level supply (2,014) was ≈350 1-g bags and 150 2-g bags of ceftriaxone, which is more than enough for a single daily dose of 1 or 2 g for the 47 inpatients

A total of 7 patients in this study were admitted to ICU at the KP San Diego Medical Center, from a population of ≈495,192 members. This number does not include patients hospitalized in non-KP facilities, patients on which complete information was not available. Assuming that the number of patients admitted to the ICU/total admissions to the hospital for ILI would be about the same for the non-KP facilities as for the KP San Diego Medical Center, since there were 81 hospitalizations for ILI in non-KP facilities, there would have been ≈6 patients admitted to non-KP ICUs during October–December 2009. Combined, these numbers would result in an estimated 13 ICU admissions/495,192 population or 26 ICU admissions/1,000,000 population. In The Australian and New Zealand Intensive Care (ANZIC) study, from June 1–August 31, 2009, a total of 28.7 cases/1 million inhabitants were admitted to the ICUs of Australia and New Zealand (*7*). A population study in Denmark found 9 (5.69%) of 158 patients were admitted to a hospital ICU during the second wave of the 2009 influenza pandemic (*8*), compared with ≈7.6% (≈13/171) in this study. 

The median LOS in ICU was 18 days (mean 18) in this study versus 7 days in the ANZIC study (*7*) and 22 days in the Orsted study (*8*). To exceed the KP San Diego Medical Center ICU bed capacity of 34 with just influenza A patients, assuming an LOS of 18, as noted above, 2 patients per day would need to be admitted for 18 consecutive days; >2 patients per day would result in exceeding the ICU bed capacity sooner. At 18 days, 36 patients would have been admitted compared to the 7 admitted in this study. Thus, a much higher attack rate would be necessary, or the number with severe disease greater, to exceed the 34-bed ICU capacity.

In this study, 56.7% patients received oxygen, 13.3% BiPAP/CPAP, and 6.7% (6/90, but 6/7 in ICU) ventilation in the KP San Diego Medical Center. If an estimate is made of patients ventilated in non-KP facilities in the same manner as that used above, an additional 5 patients would have been ventilated, for a total of 11 ventilated patients/495,192 population or ≈22 ventilated patients/1 million population. For comparison, the rate in the ANZIC study was 18 ventilated patients/1 million population (*7*). The median LOS on a ventilator in this study was 13 (mean 14.7) days, compared with 8 days in the ANZIC study (*7*). In a study of critically ill patients with influenza A(H1N1)pdm09, Kumar et al. found that 81% of patients required ventilation; the median LOS on a ventilator in that study was 12 days (*9*). In the Orsted study, the median LOS on a ventilator was 17 days (*8*). Regarding vasopressors, in this study, 6 (85.7%) of 7 patients admitted to the ICU required vasopressors for a median duration of 3 days. In the ANZIC study, 498/722 (≈69%) were provided vasopressor support (*7*).

Table 3 shows data on inpatients with pneumonia, sorted by month and facility. The complete data of clinical diagnosis of pneumonia versus a radiologist’s diagnosis of pneumonia on the basis of chest radiograph was not available for patients cared for outside the KP network. Data extracted from the KP San Diego Medical Center charts is sorted by ILI diagnosis, clinical diagnosis of pneumonia, and a radiologist’s diagnosis of pneumonia on the basis of chest radiograph. The mean monthly LOS for patients with ILI was 4, 3.5, and 2.7 days and that for a radiologist’s diagnosis of pneumonia on the basis of chest radiograph was 12.7, 8.5, and 4 for the months of October, November, and December, respectively.

## Conclusions

In conclusion, this study of a stable population during the second wave of the 2009 influenza pandemic provides good estimates of the number of patients who accessed outpatient care for ILI and those admitted to the hospital. Outpatient treatment data includes antimicrobial therapy of those with and without pneumonia. Inpatient treatment data includes the treatment of those with and without pneumonia, and the level of care (medical bed, telemetry bed, ICU), respiratory therapy (oxygen, BIPAP/CPAP, ventilation), antimicrobial therapy, vasopressors, and hemodialysis. The comparisons made with data from this and other studies are surprisingly similar. This data can be used to improve epidemiologic models, although it is anticipated that these models will need revision over time, just as the FluSurge program has been revised, to account for anticipated changes in characteristics of influenza A, population demographics, and medical therapeutics.

Technical Appendix Demographic data of study populations, diagnostic data and criteria, and health care methods provided in outpatient and inpatient facilities during the 2009 Influenza A(H1N1) pandemic in San Diego, California, USA.
