# Supplementary material for: Population Effects of Influenza A(H1N1) Pandemic among Health Plan Members, San Diego, California, USA, October–December 2009
Source: Emerg Infect Dis. 2016 Feb;22(2):255–60. doi: 10.3201/eid2202.150618 (PMC4734517; doi:10.3201/eid2202.150618)
Supplement: Technical Appendix — Demographic data of study populations, diagnostic data and criteria, and health care methods provided in outpatient and inpatient facilities during the 2009 Influenza A(H1N1) pandemic in San Diego, California, USA. [file 15-0618-Techapp-s1.pdf]

Article DOI: http://dx.doi.org/10.3201/eid2202.150618

Profile of the 2009 Influenza A (H1N1) Pandemic among Health Maintenance Organization Members, San Diego, California, USA

Technical Appendix

Technical Appendix Table 1. Demographic data of Kaiser Permanente San Diego members, and of San Diego County residents not enrolled in Kaiser Permanente plans, California, USA, 2009

Characteristics	Kaiser Permanente	San Diego County
Age	Percent	Percent
0 to 4 Years	5.2	7.25
5 to 14 Years	12	12.67
15 to 24 Years	13	15.67
25 to 44 Years	25.7	28.57
45 to 64 Years	30.7	24.28
65+ Years	13.3	11.56
Sex		
Male	48.2	49.89
Female	51.8	50.11
Race/ethnicity		
White	45.63	49.78
Hispanic	26.68	30.21
Black	5.07	5.22
Asian/Pacific Islander*	8.16	10.65
Other	1.5	4.14
Unknown	12.96	ND
Spoken language		
English	87.06	64.64
Spanish	9.03	10.93
Asian/Pacific Island languages	0.55	3.41
Other	3.36	1.27
Bilingual	ND	20.41
Household income, annual		
<$45,000	21.9	48.34
$45,000–$75,000	46.84	24.88
$75,000–$100,000	31.15	11.42
$100,000–$125,000	0.11	6.46
>$125,000	ND	8.91
Completed education		
< High School Graduate	6.48	14.82
High School Graduate	17.61	20.17
Some college or associate degree	75.8	30.99
Bachelor’s degree	ND	21.32
Graduate degree	ND	12.69
No data	0.11	NA
Body mass index category		
No data	29.65	NA
0–18.49 (underweight)	7.45	2.2
18.5–24.99 (normal)	22.61	42.5
25.0–29.99 (overweight)	21.22	33.4
30.0 or higher (obese)	19.06	21.9
Cigarette smoking		
No data	44.32	.ND

Characteristics	Kaiser Permanente	San Diego County
Current	6.86	11.8
Former	8.37	.ND
Never	37.76	.ND
Unknown	2.68	ND
Not currently	ND	88.2

Sources of San Diego County data:
County of San Diego, Health and Human Services Agency, Public Health Services, Community Health Statistics Unit, 2015; the San Diego County Demographic Profiles, 2009; retrieved 08/10/2015 from www.SDHealthStatistics.com
Spoken language: San Diego County American Community Survey from the US Census Bureau.
San Diego household income: Living Well San Diego, a county health initiative.
San Diego Education ACS and Living Well San Diego.
San Diego County obesity respondents 18 years old or older were asked for their height and weight and the body mass index was calculated by a county employee.
San Diego County smoking respondents were asked a series of smoking-related questions

Technical Appendix Table 2. Chronic diagnoses tracked by Kaiser Permanente for members and tracked by San Diego County for non-member County residents, California, USA

Kaiser Permanente members		Non-Kaiser Permanente members		
Diagnosis	%	Year dx	%	Sample Population
Asthma	4.8	2009	12.3	375,000 (SDC)
Coronary artery disease	4.0	2009	6.4	146,000 (SDC)
Chronic kidney disease	1.7	2012*	14	NHANES (USA)
Cardiovascular disease	10.0	ND	ND	ND
Diabetes mellitius	6.4	2009	7.8	178,000 (SDC)
Hypertension	20.7	2009	26.3	599,000 (SDC)

SDC, San Diego County; NHANES, National Health and Nutrition Examination Survey; ND, do data.
San Diego County data was acquired by a survey. Respondents were asked:
Asthma "Has a doctor ever told you that you have asthma?"
Coronary artery disease "Has a doctor ever told you that you have any kind of heart disease?"
Diabetes mellitus “Other than during pregnancy, had/has a doctor ever told you that you have diabetes?”
*Chronic kidney disease; data from 2012 United States Renal Data System report (http://www.usrds.org/atlas12.aspx)

Diagnostic Criteria

Kaiser Permanente criteria for the following diagnoses included in Technical Appendix Table 2: Asthma, Atherosclerotic Cardiovascular Disease, Heart Failure (HF), Chronic Kidney Disease (CKD), Cardiovascular Disease, Diabetes, Hypertension.

Asthma

Inclusion Criteria

Current KP members 5 years old and over who meet at >1 of the following criteria using a 12-month rolling window:

• Any hospital discharge *ICD-9* code, ED visit, OR outpatient diagnosis code of 493.xx excluding 493.2x (asthma)

• ≥ 2 or more dispensing records for an inhaled steroid, inhaled or oral beta-agonist, inhaled or oral anti-inflammatory agent for asthma, or bronchodilator

• Manually added to the asthma population using the Modify Population Status (MPS) tool

Atherosclerotic Cardiovascular Disease (ASCVD)

Patient Identification

A. Any discharge hospital ICD-9 code of OR ≥2 outpatient visits within a two-year window of each other with diagnosis codes of:

• 410.xx (acute myocardial infarction)

• 411.xx (other acute and subacute forms of ischemic heart disease)

• 412.xx (old myocardial infarction)

• 413.xx (angina pectoris) excluding 413.1 (prinzmetal angina)

• 414.xx (other forms of chronic ischemic heart disease) excluding 414.1 (aneurysm and dissection of heart)

• 433.xx (occlusion and stenosis of precerebral arteries)

• 437.1 (other generalized ischemic cerebrovascular disease)

• 440.1 (atherosclerosis of renal artery)

• 440.2 (atherosclerosis native art extremities unspec)

• 440.21 (atheroslero native art extreme w/intermit claudicat)

• 440.22 (atheroslero native art extremities w/rest pain)

• 440.23(atheroslero native art extremities w/ulceration)

• 440.24 (atheroslero native art extremities w/gangrene)

• 440.29 (other atherosclerosis of native arteries of the extremities)

• 440.3 (atherosclerosis unspec bypass graft extremities)

• 440.31 (atherosclerosis autol vein byps graft extremities)

• 440.32 (atherosclerosis nonautol biologic byps gft extrem)

• 440.4 (chronic total occlusion artery extremities)

• 441.xx (aortic aneurysm and dissection)

• 443.9x (peripheral vascular disease, unspecified)

• 444.0 (arterial embolism and thrombosis)

• 445.xx (atheroembolism)

• V45.81 (aortocoronary bypass surgical status)

• V45.82 (percutaneous transluminal coronary angioplasty surgical status)

B. Any procedure ICD-9 code of:

• 36.0× (removal of coronary artery obstruction and insertion of stent(s))

• 36.1× (bypass anastomosis for heart revascularization)

• 36.2 (heart revascularization by arterial implant)

• 00.66 (percutaneous transluminal coronary angioplasty [PTCA] or coronary atherectomy)

Heart Failure

Patient Identification

Current KP members 18 and older who meet ≥1 of the following criteria:

A. Coded with ≥1 ICD-9 diagnosis code from a hospital discharge, outpatient encounter or an active problem at a Kaiser facility within a rolling 3-year period from Table 1.

B. Coded with ≥2 ICD-9 diagnosis codes found Table 2 from the claims data within a rolling 3-year period.

C. Placed on the HF -patient-addition list.

Identification ICD-9 Codes

ICD-9 Code	Description
402.01	MALIG HYPERTENSIVE HEART DISEASE W/HEART FAILURE
402.11	BEN HTN HEART DISEASE WITH HEART FAIL
402.91	UNSPEC HYPERTENSIVE HEART DISEASE W/HEART FAIL
425.2	OBSCURE CARDIOMYOPATHY OF AFRICA
425.5	ALCOHOLIC CARDIOMYOPATHY
425.7	NUTRITIONAL AND METABOLIC CARDIOMYOPATHY
425.8	CARDIOMYOPATHY OTHER DISEASES CLASSIFIED ELSW
425.9	UNSPECIFIED SECONDARY CARDIOMYOPATHY
402.11C	BENIGN HYPERTENSIVE HEART DISEASE W HEART FAILURE
402.91C	HYPERTENSIVE HEART DISEASE W HEART FAILURE
402.9D	HYPERTENSIVE CARDIOMYOPATHY
404.01B	MALIGNANT HYPERTENSIVE HEART AND RENAL DISEASE W HEART FAILURE
404.03B	MALIGNANT HYPERTENSIVE HEART AND RENAL DISEASE W HEART AND RENAL FAILURE
404.11A	BENIGN HYPERTENSIVE HEART AND RENAL DISEASE WITH CHF (inactive).
404.11B	BENIGN HYPERTENSIVE HEART AND RENAL DISEASE W CHF
404.13A	BENIGN HYPERTENSIVE HEART AND RENAL DISEASE WITH CHF RENAL FAIL(INACTIVE).
404.13B	BENIGN HYPERTENSIVE HEART AND RENAL DISEASE W CHF AND RENAL FAILURE
404.13C	BENIGN HYPERTENSIVE HEART AND RENAL DISEASE W CHF AND RENAL FAILURE.
404.13D	BENIGN HYPERTENSIVE HEART AND RENAL DISEASE W HEART AND RENAL FAILURE
414.8A	ISCHEMIC CARDIOMYOPATHY
414.8D	CARDIOMYOPATHY, DILATED, ISCHEMIC
425.2A	CARDIOMYOPATHY, OBSCURE AFRICAN
425.4A	CARDIOMYOPATHY, PRIMARY
425.4B	CARDIOMYOPATHY
425.4C	CARDIOMYOPATHY, COEXISTENT W OTHER DISEASE
425.4D	CARDIOMYOPATHY, IDIOPATHIC
425.4E	CARDIOMYOPATHY, NONOBSTRUCTIVE
425.4M	NONISCHEMIC DILATED CARDIOMYOPATHY
425.5A	CARDIOMYOPATHY, DILATED, DUE TO ALCOHOL
425.5B	CARDIOMYOPATHY, DUE TO COBALT
425.7B	CARDIOMYOPATHY, DUE TO NUTRITIONAL OR METABOLIC DISORDER
425.8E	CARDIOMYOPATHY IN DISEASE
425.9A	CARDIOMYOPATHY, DILATED, DUE TO DRUG
425.9C	CARDIOMYOPATHY, DILATED, DUE TO TOXIC REACTION.
425.9E	CARDIOMYOPATHY, SECONDARY
425A	CARDIOMYOPATHY.
428.0A	CHF
428.0B	CHF, ACUTE
428.0E	CHF, W RIGHT HEART FAILURE
428.0F	CHF, CHRONIC
428.0G	CHF, STAGE D
428.0H	CHF, STAGE C
428.0I	CHF, STAGE B
428.0J	CHF, STAGE A
428.0K	CHF, CHRONIC, STAGE D
428.0L	CHF, CHRONIC, STAGE C
428.0M	CHF, CHRONIC, STAGE B
428.0N	CHF, CHRONIC, STAGE A
428.0V	CONGESTIVE HEART FAILURE: (CHF).
428.0W	CHF EXACERBATION
428.1A	LEFT HEART FAILURE
428.1B	HEART FAILURE, LT SIDE W/LVEF less than or equal to 40%
428.1C	HEART FAILURE, LT SIDE W/LVEF >40%.
428.1D	HEART FAILURE, LT SIDE W/LVEF UNKNOWN.
428.1E	CHF W LEFT VENTRICULAR SYSTOLIC DYSFUNCTION

ICD-9 Code	Description
428.1F	LEFT HEART FAILURE W LVEF 0.3 OR LESS
428.1G	HEART FAILURE, RT SIDED, ISOLATED
428.1H	HEART FAILURE, LT SIDE W LVEF 31-40%
428.1I	HEART FAILURE, LT SIDE W LVEF less than 30%
428.20A	SYSTOLIC DYSFUNCTION.
428.20D	SYSTOLIC HEART FAILURE
428.21A	LEFT VENTRICULAR SYSTOLIC HEART FAILURE, ACUTE
428.21B	SYSTOLIC HEART FAILURE, ACUTE
428.22A	LEFT VENTRICULAR SYSTOLIC DYSFUNCTION, CHRONIC.
428.22B	SYSTOLIC HEART FAILURE, CHRONIC
428.23A	LEFT VENTRICULAR SYSTOLIC HEART FAILURE, ACUTE ON CHRONIC
428.23B	SYSTOLIC HEART FAILURE, ACUTE ON CHRONIC
428.30B	DIASTOLIC HEART FAILURE
428.31A	DIASTOLIC HEART FAILURE, ACUTE
428.32A	DIASTOLIC HEART FAILURE, CHRONIC
428.33A	DIASTOLIC HEART FAILURE, ACUTE ON CHRONIC
428.40A	COMBINED SYSTOLIC AND DIASTOLIC DYSFUNCTION
428.41A	COMBINED SYSTOLIC AND DIASTOLIC HEART FAILURE, ACUTE
428.42A	COMBINED SYSTOLIC AND DIASTOLIC HEART FAILURE, CHRONIC
428.43A	COMBINED SYSTOLIC AND DIASTOLIC HEART FAILURE, ACUTE ON CHRONIC
428.9B	HEART FAILURE
428.9C	NEW YORK HEART FAILURE CLASS 1
428.9D	NEW YORK HEART FAILURE CLASS 2
428.9E	NEW YORK HEART FAILURE CLASS 3
428.9F	NEW YORK HEART FAILURE CLASS 4
428.9G	NEW YORK HEART FAILURE CLASS INDETERMINATE
428.9H	HEART FAILURE STAGE D
428.9I	HEART FAILURE STAGE A
428.9J	HEART FAILURE, STAGE C
428.9K	HEART FAILURE, STAGE B
428.9O	LOW CARDIAC OUTPUT SYNDROME
429.3F	LEFT VENTRICULAR DILATATION
429.3H	CARDIOMYOPATHY, DILATED
429.4H	CARDIAC INSUFFICIENCY AFTER CARDIAC SURGERY, LATE POSTOP COMPLICATION.
429.89B	LEFT VENTRICULAR SYSTOLIC DYSFUNCTION, CHRONIC
429.9F	SYSTOLIC DYSFUNCTION, LEFT VENTRICLE
500688	HYPERTENSIVE CHF
500693	HYPERTENSIVE HEART AND RENAL DISEASE W CHF
500696	CHF DUE TO VALVULAR DISEASE
500705	BENIGN HYPERTENSIVE HEART DISEASE W CHF
500706	MALIGNANT HYPERTENSIVE HEART AND RENAL DISEASE W CHF
500707	MALIGNANT HYPERTENSIVE HEART AND RENAL DISEASE W CHF AND RENAL FAILURE
500708	HYPERTENSIVE HEART AND RENAL DISEASE W HEART AND RENAL FAILURE
500969	MALIGNANT HYPERTENSIVE HEART DISEASE W CHF.
501317	HYPERTENSIVE KIDNEY AND HEART DISEASE, W HEART FAILURE, ESRD, ON DIALYSIS
501318	HYPERTENSIVE KIDNEY AND HEART DISEASE, W HEART FAILURE, CKD 5
501319	HYPERTENSIVE KIDNEY AND HEART DISEASE, W HEART FAILURE, CKD 4
501320	HYPERTENSIVE KIDNEY AND HEART DISEASE, W HEART FAILURE, CKD 3
501321	HYPERTENSIVE KIDNEY AND HEART DISEASE, W HEART FAILURE, CKD 2
501322	HYPERTENSIVE KIDNEY AND HEART DISEASE, W HEART FAILURE, CKD 1
501422	BIVENTRICULAR CONGESTIVE HEART FAILURE
501500	CONGESTIVE HEART FAILURE WITH CARDIOMYOPATHY
502784	LEFT HEART FAILURE, SYSTOLIC DYSFUNCTION W LVEF 41-49%
502785	LEFT HEART FAILURE, SYSTOLIC DYSFUNCTION W LVEF 31-40%
502786	LEFT HEART FAILURE, SYSTOLIC DYSFUNCTION W LVEF 0.3 OR LESS
502787	LEFT HEART FAILURE, SYSTOLIC DYSFUNCTION W LVEF 0.5 OR GREATER

Claims Identification ICD-9 Codes

ICD-9 Code	Description
402.01	MALIGNANT HYPERTENSIVE HEART DISEASE WITH HEART FAILURE
402.91	UNSPECIFIED HYPERTENSIVE HEART DISEASE WITH HEART FAILURE
404.01	HYPERTENSIVE HEART AND CHRONIC KIDNEY DISEASE MALIGNANT WITH HEART FAILURE WITH CHRONIC KIDNEY DISEASE
404.03	HYPERTENSIVE HEART AND CHRONIC KIDNEY DISEASE MALIGNANT WITH HEART FAILURE WITH CHRONIC KIDNEY DISEASE
404.11	HYPERTENSIVE HEART AND CHRONIC KIDNEY DISEASE BENIGN WITH HEART FAILURE WITH CHRONIC KIDNEY DISEASE
404.13	HYPERTENSIVE HEART AND CHRONIC KIDNEY DISEASE BENIGN WITH HEART FAILURE WITH CHRONIC KIDNEY DISEASE
425.2	OBSCURE CARDIOMYOPATHY OF AFRICA
425.4	OTHER PRIMARY CARDIOMYOPATHIES
425.5	ALCOHOLIC CARDIOMYOPATHY
425.7	NUTRITIONAL AND METABOLIC CARDIOMYOPATHY
425.8	CARDIOMYOPATHY IN OTHER DISEASES CLASSIFIED ELSEWHERE
425.9	SECONDARY CARDIOMYOPATHY UNSPECIFIED
428	CONGESTIVE HEART FAILURE UNSPECIFIED
428.1	LEFT HEART FAILURE
428.2	UNSPECIFIED SYSTOLIC HEART FAILURE
428.21	ACUTE SYSTOLIC HEART FAILURE
428.22	CHRONIC SYSTOLIC HEART FAILURE
428.23	ACUTE ON CHRONIC SYSTOLIC HEART FAILURE
428.3	UNSPECIFIED DIASTOLIC HEART FAILURE
428.31	ACUTE DIASTOLIC HEART FAILURE
428.32	CHRONIC DIASTOLIC HEART FAILURE
428.33	ACUTE ON CHRONIC DIASTOLIC HEART FAILURE
428.4	UNSPECIFIED COMBINED SYSTOLIC AND DIASTOLIC HEART FAILURE
428.41	ACUTE COMBINED SYSTOLIC AND DIASTOLIC HEART FAILURE
428.42	CHRONIC COMBINED SYSTOLIC AND DIASTOLIC HEART FAILURE
428.43	ACUTE ON CHRONIC COMBINED SYSTOLIC AND DIASTOLIC HEART FAILURE
428.9	HEART FAILURE UNSPECIFIED
429.4	FUNCTIONAL DISTURBANCES FOLLOWING CARDIAC SURGERY
429.89	OTHER ILL-DEFINED HEART DISEASES
500688	HYPERTENSIVE CHF
500693	HYPERTENSIVE HEART AND RENAL DISEASE W CHF
500696	CHF DUE TO VALVULAR DISEASE
500705	BENIGN HYPERTENSIVE HEART DISEASE W CHF
500706	MALIGNANT HYPERTENSIVE HEART AND RENAL DISEASE W CHF
500707	MALIGNANT HYPERTENSIVE HEART AND RENAL DISEASE W CHF AND RENAL FAILURE
500708	HYPERTENSIVE HEART AND RENAL DISEASE W HEART AND RENAL FAILURE
500969	MALIGNANT HYPERTENSIVE HEART DISEASE W CHF.
501317	HYPERTENSIVE KIDNEY AND HEART DISEASE, W HEART FAILURE, ESRD, ON DIALYSIS
501318	HYPERTENSIVE KIDNEY AND HEART DISEASE, W HEART FAILURE, CKD 5
501319	HYPERTENSIVE KIDNEY AND HEART DISEASE, W HEART FAILURE, CKD 4
501320	HYPERTENSIVE KIDNEY AND HEART DISEASE, W HEART FAILURE, CKD 3
501321	HYPERTENSIVE KIDNEY AND HEART DISEASE, W HEART FAILURE, CKD 2
501322	HYPERTENSIVE KIDNEY AND HEART DISEASE, W HEART FAILURE, CKD 1
501422	BIVENTRICULAR CONGESTIVE HEART FAILURE
501500	CONGESTIVE HEART FAILURE WITH CARDIOMYOPATHY

Chronic Kidney Disease

Inclusion Criteria

The identification algorithm for chronic kidney disease is based on member age, gender, race and laboratory results for creatinine and urine protein/microalbumin. The staging used in the algorithm is based on K/DOQI guidelines for Stages 1–5. Inclusion is based on meeting the rules

for Stages 1–5 as defined below. Dialysis and transplant patients are captured within CKD as separate sub-stages.

- Patient age ≥ 18 AND
- Meets the criteria for one of the stages

Stages of Chronic Kidney Disease (CKD)

Staging of CKD is based on *GFR* Stage 1–3 have additional requirements as noted below.

CKD<Stage (patients)	GFR ml/min/1.73 m^2 BSA	Description
1[=]	> 90	Normal GFR
2[=]	60-89[§]	Mild ↓ GFR
3*	30-59[§]	Moderate ↓ GFR
4	15-29	Severe ↓ GFR
5	<15	Kidney Failure

[<] Chronic is defined as persisting for 3 months or more. The 2 GFR values that are used are the last result and the most recent result that was collected at least 90 days before the last was collected. There is no maximum time between the 2 results.

[=] Stage 1 and 2 also require a marker of kidney disease: proteinuria, hematuria or an anatomic abnormality are outlined in K/DOQI. In POINT, macroscopic proteinuria is used as the marker. Macroscopic proteinuria is defined as total urine protein or MAU > 300 OR protein to creatinine ratio *1000 > 200. There must be 2 consecutive results. There must be ≥1 day between sample collections. Urine samples obtained during a pregnancy and up to 3 months after pregnancy are not evaluated for the proteinuria requirement.

= Stage 1 and 2 can also be flagged for staging within the Renal Population Tool. If this flag is set, the member would not require the macroscopic protein requirement.

* In POINT, Stage 3 is limited to patients at highest risk of ESRD and must have one or more additional marker aside from the GFR within the 30–59 range:

- macroscopic proteinuria
- (½ age + GFR) < 85
- Member marked for staging within the Renal Population Tool

§ Patients with GFR in these ranges without other markers or risk factors are considered to be at lower risk for ESRD.

CKD Sub-levels were added to CKD 3 and 4 to further stratify members. The sub-levels follow the same rules for initial and restaging as set for all other CKD stages. The higher level stages of 3 and 4 continue to be assigned to members with the addition of the sub-levels for use when appropriate.

STAGE	SUBSTAGE	MIN GFR	MAX GFR
CKD Stage 3	A	45	59
	B	30	44
CKD Stage 4	A	25	29
	B	20	24
	C	15	19

Dialysis patients are identified by the assigned modality in the Renal Population Tool. Members are either assigned to a stage of Hemodialysis (HEMO) or Peritoneal Dialysis (PD) within CKD Care Management. If a member is identified as dialysis, that is the default stage.

Kidney transplant patients are identified by the modality or transplant status of transplanted within the Renal Population Tool. Members are assigned to substages (TP SUBSTAGE 1–5) based on GFR ranges within CKD Care Management.

Kidney transplant patients are identified by a report that is provided to CarePOINT. Members are assigned to substages (TP SUBSTAGE 1–5) based on GFR ranges within CKD Care Management.

Cardiovascular Disease

Patient Identification

The CVD Cardiovascular Disease population is currently comprised of members from the following POINT registries:

• Diabetes

• Coronary artery disease

• Heart failure

• Chronic kidney disease (excluding kidney transplant patients unless they are in one or more of the previously mentioned populations)

For patient identification criteria, please refer to the individual conditions cited above.

Diabetes

Inclusion Criteria

Current KP members 18 and older who meet ≥1 of the following criteria:

A. Two or more outpatient ICD-9 diagnosis codes from Table 1 since 2005 (excludes claims and outpatient codes from ER, Obstetrics/Gynecology, Podiatry, and Ophthalmology – these patients are placed in DM Unverified)

B. Have at least one outpatient ICD-9 diagnosis code since 2005 (excludes claims and outpatient codes from ER, Obstetrics/Gynecology, Podiatry, and Ophthalmology – these patients are placed in DM Unverified) from Table 1 and meet ≥1 of the following criteria:

I. Any history of hemoglobin A1C > 7.5% or fructosamine > 319 µmoL

II. Last 2 A1Cs >= 6.5% in the past 24 months

III. A dispensing record of an oral hypoglycemic in Table 2 (excludes metformin, exenatide, pioglitazone, rosiglitazone, or repaglinide only) or insulin since 2005

IV. Any history of more than one FBS > 126 mg/dL and patient has dispensing record of one of the above excluded medications since 2005

C. Manually added to the diabetes population using the Modify Population Status (MPS) tool

D. Active Dx code on the Problem List AND ≥1 outpatient ICD-9 code since 2005 (includes claims and outpatient codes from ER, Obstetrics/Gynecology, Podiatry, and Ophthalmology)

Hypertension

Patient Identification

Current KP members 18 and older who meet ≥1 of the following criteria:

A. Two outpatient visits within 365 days of each other with a diagnosis code for hypertension

B. One outpatient visit with a diagnosis code for hypertension one hospital discharge with a diagnosis code for hypertension within 365 days of each other

C. One antihypertensive dispensing in the past 6 months and 1 outpatient visit with a hypertension diagnosis code within 365 days of the dispense date

D. One outpatient visit with a code for hypertension AND a member of 1 of the following POINT Population Care Management (PCM) populations:

- Heart Failure
- CAD
- Diabetes
- CKD
- CVA (excluding subarachnoid, subdural and cardioembolic)

E. Manually added to the Hypertension population using the Modify Population Status (MPS) tool

Technical Appendix Table 3. Combinations of selected chronic conditions among Kaiser Permanente Health Plan Members, San Diego, California, USA, December 2009

Asthma	CAD	CKD	CVD	DM	HTN	Frequency
N	N	N	N	N	N	111,075
N	N	N	N	N	Y	58,667
N	N	N	Y	N	N	223
N	N	N	Y	N	Y	1,097
N	N	N	Y	Y	N	4,803
N	N	N	Y	Y	Y	17,043
N	N	Y	N	N	N	7
N	N	Y	N	N	Y	100
N	N	Y	Y	N	N	546
N	N	Y	Y	N	Y	2,264
N	N	Y	Y	Y	N	104
N	N	Y	Y	Y	Y	2,415
N	Y	N	Y	N	N	1,964
N	Y	N	Y	N	Y	10,528
N	Y	N	Y	Y	N	182
N	Y	N	Y	Y	Y	4,340
N	Y	Y	Y	N	N	23
N	Y	Y	Y	N	Y	1,021
N	Y	Y	Y	Y	N	11
N	Y	Y	Y	Y	Y	1,699
Y	N	N	ND	N	N	19,893
Y	N	N	ND	N	Y	2,366
Y	N	N	Y	N	N	10
Y	N	N	Y	N	Y	26
Y	N	N	Y	Y	N	236
Y	N	N	Y	Y	Y	639
Y	N	Y	N	N	Y	6
Y	N	Y	Y	N	N	33
Y	N	Y	Y	N	Y	85
Y	N	Y	Y	Y	N	6
Y	N	Y	Y	Y	Y	93
Y	Y	N	Y	N	N	32
Y	Y	N	Y	N	Y	103
Y	Y	N	Y	Y	N	2
Y	Y	N	Y	Y	Y	51
Y	Y	Y	Y	N	Y	5

Asthma	CAD	CKD	CVD	DM	HTN	Frequency
Y	Y	Y	Y	Y	Y	27

Y, present; N, not present; CAD, coronary artery disease; CKD, chronic kidney disease; CVD, cardiovascular disease; DM, diabetes mellitus; HTN, hypertension, ND, no data..

Technical Appendix Table 4. Kaiser Permanente San Diego, California, USA health plan members diagnosed with influenza-like illness and pneumonia October–December, 2009, by age and gender

Age	Oct, ILI, total	Oct, ILI, male	Nov, ILI, total	Nov, ILI , male	Dec, ILI, total	Dec, ILI, male	Oct–Dec no. with pneum	Oct–Dec no. with pneum conf. by CXR, total	Oct–Dec no. with pneum conf. by CXR, male
0–4	228	134	270	149	72	36	47	25	12
5–9	312	178	380	205	62	34	40	15	12
10–14	410	216	387	199	64	36	33	11	5
15–19	297	152	316	128	78	33	16	9	3
20–24	169	63	266	97	80	36	5	1	0
25–29	140	66	240	85	73	26	5	3	2
30–34	131	44	208	75	89	37	6	3	3
35–39	116	53	209	88	76	34	11	6	3
40–44	120	53	192	69	64	19	9	7	1
46–49	104	35	179	78	81	29	6	5	2
50–54	138	49	196	70	94	35	19	14	9
55–59	77	24	123	44	68	21	7	2	1
60–64	40	15	63	21	31	12	5	2	1
65–69	21	8	37	17	24	7	4	1	1
70–74	17	5	17	6	22	6	2	1	0
75–79	7	1	13	5	11	3	3	0	0
80–84	7	2	9	4	9	2	1	0	0
85–89	3	1	2	0	4	1	0	0	0
>90	2	2	2	1	1	0	0	0	0
NA	2,339	1,101	3,109	1,341	1,003	407	219	105	55
0–18	NA	NA	NA	NA	NA	NA	136	60	32
19–>90	NA	NA	NA	NA	NA	NA	83	45	23
OOPC	93	NA	93	NA	35	NA	60	NA	NA
Total	2432	NA	3202	NA	1038	NA	NA	NA	NA
% of membership	2,432/495,718 = 0.49%	NA	3202/494,911 = 0.64%	NA	1038/494,947 = 0.21%	NA	NA	NA	NA

ILI, influenza-like illness; pneum, pneumonia; dx, diagnosis; CXR chest x-ray; OOPC, out-of-plan claims (ILI + pneumonia); NA, not applicable.

Technical Appendix Table 5. Antimicrobial regimens prescribed for outpatients diagnosed with ILI or ILI and pneumonia evaluated in a Kaiser Permanente facility October–December, 2009, San Diego, California, USA

Medication	Age, y 0–18	Age, y 19–≥90
Amoxicillin	47	7
Amoxicillin, Doxycycline	1	2
Amoxicillin/clavulanate	6	0
Amoxicillin, Azithromycin	1	1
Azithromycin	60	12
Azithromycin, Amoxicillin/Clavulanate	2	0
Azithromycin, Doxycycline	0	1
Azithromycin, Cephalexin	1	0
Azithromycin, Cefuroxime	0	2
Moxifloxacin	2	30
Doxycycline	0	12
Doxycycline, Cefuroxime	0	6
Doxycycline, Cephalexin	0	1
Doxycycline, Cefuroxime, Cephalexin, Cotrimoxazole	0	1
Cefuroxime	0	4
Cefdinir	3	0
Ciprofloxacin	0	1
None	2	3
Admitted to Hospital	6	3
Oseltamivir	104	46

Technical Appendix Table 6. Age and gender of 90 inpatients admitted to Kaiser Permanente Medical Center, San Diego, California, USA, October–December, 2009

Age	Sex, M	Sex, F	Total
0–4	4	4	8
5–9	5	3	8
10–14	1	2	3
15–19	1	4	5
20–24	2	2	4
25–29	0	5	5
30–34	1	4	5
35–39	5	3	8
40–44	2	5	7
45–49	1	3	4
50–54	6	4	10
55–59	3	6	9
60–64	2	6	8
65–69	2	0	2
70–74	0	0	0
75–79	0	0	0
80–84	1	2	3
85–89	1	0	1
>90	37	53	90

Technical Appendix Table 7. Tests for influenza A among 90 inpatients admitted to Kaiser Permanente Medical Center, San Diego October, November, December, 2009

Any test + for influenza A	PCR +	All tests – for influenza A	PCR –	Testing not done for influenza A	Culture+/PCR not done	Rapid test +/PCR not done	Total
58	NA	25	NA	7	NA	NA	90
NA	55 (5 cx +)	25	23	7	1	2	90

NA, not applicable.

Technical Appendix Table 8. Antimicrobial regimens for 90 inpatients treated in Kaiser Permanente Medical Center, San Diego, California, USA

Antibacterial drugs prescribed	No.
None	18
Ceftri, Doxy	17
Ceftri, Azithro	13
Ceftri	7
Ceftri,Vanc	1
Ceftri, Mox	1
Ceftri, Erythro	1
Ceftri, Azithro, Vanc, Metro	1
Ceftri, Azithro, P/T, Vanc	1
Ceftri,Azithro, Flucon, Vanc	1
Ceftri, Doxy, Flucon, T/S	1
Ceftri, Doxy Vanc, Aztreo	1
Ceftri, Doxy, Cipro, Metro	1
Ceftri, Ceph	1
Azithro, Doxy	1
Moxi	5
Azithro	3
Azithro, Cipro, Ceftaz	1
Azithro, Cipro, P/T, Flucon, Vanc	1
Cipro	1
Cipro, Ceftaz	1
Cipro, Vanc, Metro	1
Cipro, Ceftaz, Vanc	1
Cipro, P/T, Vanc	1
Cipro, Ceftaz, Moxi, Vanc,	1
Vanc	1
Vanco, Clinda	1
Vanc, P/T, Flucon, , Primaq, Clinda	1
Mero	2
Ceph	1
Doxy	1
Cefdinir	1

Antibacterial drugs prescribed	No.
Antivirals	NA
Oseltamivir	87
Peramivir	1
Corticosteroids	12

Ceftri ceftriaxone, Azithro, azithromycin; P/T, piperacillin/tazobactam; Vanc, vancomycin; Doxy, doxycycline; Mox, moxifloxacin; Erythro, erythromycin; Metro, metronidazole; Flucon, fluconazole; T/S, trimethoprim/Sulfamethoxazole; Aztreo, aztreonam; Ceph, cephalexin; Cipro, ciprofloxacin; Ceftaz, ceftazidime; Clinda, clindamycin; Primaq, primaquine; Mero, meropenem; NA, not applicable.

Technical Appendix Table 9. Pneumonia and tests for influenza in inpatients seen at Kaiser Permanente, San Diego, California, USA facility

Age, y	CXR, pneum	CXR, no pneum	CXR ND	CXR, Pneum, Influ A test +\-\not done	CXR, no Pneum Influ A test +\-\not done	CXR ND; Influ A test +\-\not done
0–18	5	16	3	5/0/0	7/7/2	3/0/0
19–100	27	36	3	23/2/2	18/16/2	2/0/1

CXR, chest x-ray; pneum, pneumonia; ND, not done; influ, influenza.

Technical Appendix Table 10. Estimated admissions to hospital of Kaiser Health Plan San Diego population by CDC Flu Surge2, (2005) and FluSurge Special Edition (2009) programs

	Week of epidemic wave											
Characteristics	1	2	3	4	5	6	7	8	9	10	11	12
Most likely weekly predicted admissions, FluSurg09	5	19	33	47	61	71	71	61	47	33	19	5
Predicted no. of patients in hospital, FluSurge09	3	14	24	35	45	52	54	48	41	31	21	11
Predicted no. of patients in ICU, FluSurge09	1	3	6	9	12	15	16	15	13	10	7	4
Predicted no. of patients on ventilators, FluSurge09	0	2	3	5	6	7	8	8	7	5	4	2
Predicted no. of deaths from influenza, FluSurge09	0	0	0	2	3	4	5	6	6	5	4	3
Minimum predicted no. of admissions, FluSurge05	4	15	26	37	49	56	56	49	37	26	15	4
Actual no. of health plan members in hospital	11	28	44	43	52	66	52	60	37	22	24	25

No., number; ICU, intensive care unit.
'FluSurge2' data based on assuming a 15% attack rate, a health plan population of 500,000, total staffed beds 392, total licensed ICU beds 34, total number of ventilators 40.
